# Illness acceptance and quality of life in amyotrophic lateral sclerosis: the role of health and environmental factors

**DOI:** 10.3389/fneur.2025.1721044

**Published:** 2026-01-12

**Authors:** Łukasz Czyżewski, Katarzyna Petrzak-Nocuń, Zuzanna Strząska-Kliś, Janusz Wyzgał, Urszula Religioni, Anna Augustynowicz, Jakub Świtalski, Łukasz Dudziński, Andrzej Silczuk

**Affiliations:** 1 Department of Geriatric Nursing, Medical University of Warsaw, Warsaw, Poland; 2 Department of Neurology, Medical University of Warsaw, Warsaw, Poland; 3 Department of Cardiothoracic and Transplantology, Medical University of Warsaw, Warsaw, Poland; 4 Department of Nephrology Nursing, Medical University of Warsaw, Warsaw, Poland; 5 School of Public Health, Centre of Postgraduate Medical Education of Warsaw, Warsaw, Poland; 6 Department of Ethics and Medical Law, Center for the Humanities and Social Sciences in Medicine, Medical University of Warsaw, Warsaw, Poland; 7 Medical Rescue Department, Medical University of Warsaw, Warsaw, Poland; 8 Department of Community Psychiatry, Medical University of Warsaw, Warsaw, Poland

**Keywords:** acceptance of illness scale, ALS, amyotrophic lateral sclerosis, health-related quality of life, HRQOL, psychological adaptation, WHOQOL-BREF

## Abstract

**Purpose:**

To determine the extent to which illness acceptance accounts for variability in health-related quality of life (HRQoL) among adults with amyotrophic lateral sclerosis (ALS) attending a hospital-based outpatient clinic, after controlling for sociodemographic and health variables.

**Materials and methods:**

We conducted a single-center, cross-sectional study in a hospital outpatient clinic. Adults with ALS completed the World Health Organization Quality of Life-BREF (WHOQOL-BREF) and the Acceptance of Illness Scale (AIS), plus a sociodemographic and health questionnaire.

**Results:**

Forty-five patients were analyzed (mean age 52 ± 14 years; 58% women). WHOQOL-BREF domain means were: physical 46.9 ± 14.1, psychological 51.2 ± 16.9, social 53.0 ± 24.6, environment 58.4 ± 18.4. Mean AIS was 20.4 ± 8.1. AIS correlated positively with all domains (r = 0.40–0.52, all *p* ≤ 0.006). In age- and sex-adjusted models, AIS independently predicted higher scores: physical *β* = 0.96 (*p* = 0.003), psychological β = 0.94 (*p* = 0.013), social β = 1.47 (*p* = 0.003), environment β = 1.10 (*p* = 0.025). Percutaneous endoscopic gastrostomy (PEG) was associated with lower physical and environment scores than oral feeding. Respiratory status differentiated physical and psychological scores. Better living conditions related to higher psychological and environment scores. Time from first symptoms to diagnosis correlated with AIS (*ρ* = 0.37, *p* = 0.014).

**Conclusion:**

Illness acceptance is a robust, independent correlate of HRQoL across domains in ALS. Care should pair symptom control with brief acceptance-focused, educational, and family communication interventions, and address environmental needs. Decisions on PEG and non-invasive ventilation (NIV) should include routine dietetic, psychological, and speech–language input. Longitudinal studies should test AIS as a mediator of somatic and environmental interventions on HRQoL.

## Introduction

Amyotrophic lateral sclerosis (ALS) is a progressive neurodegenerative disease in which motor and respiratory disability advances relatively fast, and survival is usually measured in years from symptom onset. In the absence of disease-modifying therapy, care focuses on maintaining independence and relieving burden, with an emphasis on health-related quality of life (HRQoL). In the World Health Organization (WHO) framework, HRQoL is multidimensional and covers physical, psychological, and social functioning in the patient’s environmental context. Population studies in ALS show that poorer functional status, bulbar phenotype, and psychological distress reduce HRQoL, whereas ventilatory support and assistive technologies can improve it at comparable disease stages. ALS-specific tools can also be more sensitive than generic measures in detecting changes in quality of life (1). Non-motor factors such as pain, anxiety-depressive symptoms, sleep disturbance, and fatigue are also linked to lower HRQoL, and their co-occurrence may cumulatively worsen HRQoL (2). The contemporary QuALS framework synthesizes living with ALS across three domains and demonstrates insufficient coverage of psychological and social components. This raises the risk of underestimating psychosocial determinants of HRQoL in research and clinical reports (3). A systematic review following Consensus-based Standards for the selection of health Measurement Instruments (COSMIN) documents uneven evidence on measurement properties of popular tools, indicating the need for better validation and more consistent reporting in ALS cohorts (4).

Current guidelines from the National Institute for Health and Care Excellence (NICE) and the European Academy of Neurology (EAN) recommend coordinated interprofessional care with regular needs assessment and early implementation of nutritional interventions, including qualification for percutaneous endoscopic gastrostomy (PEG), and respiratory interventions, including non-invasive ventilation (NIV). They also highlight the importance of supportive communication for symptom management and the measurement of outcomes that matter to patients, including HRQoL (5, 6). In parallel, updated COSMIN guidance recommends rigorous evaluation of measurement properties and transparent reporting of certainty of evidence, which supports careful selection and interpretation of patient-reported outcome measures (PROMs) (7).

Given the multidimensional nature of HRQoL and the heterogeneity of patient-reported outcomes at comparable clinical stages, identifying potentially modifiable determinants, particularly psychological resources, may help explain between-patient variability and inform supportive care (8). Illness acceptance, within the illness-cognitions/appraisals framework, can be viewed as the degree to which an individual comes to terms with illness-related limitations and integrates them into everyday life; conceptually, higher acceptance reflects better psychological adaptation and may be associated with lower emotional distress (9). The construct can be operationalized with the 8-item Acceptance of Illness Scale (AIS), which captures perceived consequences of poor health (e.g., limitations, dependence on others, reduced self-esteem); total scores range from 8 to 40, with higher scores indicating greater acceptance (10). While ALS-specific psychometric validation of the AIS remains scarce, a related 8-item acceptance measure from the Felton–Revenson tradition has been administered in an ALS cohort (reported as AOI), documenting prior questionnaire-based assessment of illness acceptance in this population (11). In ALS, subjective well-being and HRQoL show substantial interindividual variability; in a highly selected locked-in state cohort, positive well-being has been reported even in the absence of residual physical function, underscoring that objective disability does not fully determine subjective outcomes (12). In broader ALS samples, functional impairment can relate to QoL and mood, but these associations appear context-dependent (e.g., moderated by country), supporting a multifactorial view that includes psychological and socio-cultural determinants (13). Consistent with this, illness cognitions assessed in the first year after ALS diagnosis (including acceptance) have been associated with HRQoL (9). Qualitative evidence further indicates that acceptance is intertwined with how people with ALS engage with supportive services and make care decisions across the life course (14). Importantly, this line of inquiry complements, rather than replaces-HRQoL measurement: validated HRQoL instruments quantify patient-reported outcomes, whereas illness acceptance represents a potentially modifiable determinant of between-patient variability in those outcomes. Against this background, data are still lacking on the role of psychological resources, especially illness acceptance, as an independent correlate of HRQoL variability in ALS after accounting for sociodemographic and clinical factors. This study addresses that gap by examining associations between illness acceptance and multidimensional WHOQOL-BREF domains while controlling for sociodemographic and clinical variables in line with current standards.

### Purpose

The aim was to assess the extent to which the level of illness acceptance explains variability in quality of life among patients with ALS seen in a hospital-based outpatient clinic, after controlling for sociodemographic and health variables.

## Materials and methods

### Study design and setting

We conducted a single-center, cross-sectional observational study at the Neurology Outpatient Clinic, Central Clinical Hospital, University Clinical Centre, Medical University of Warsaw, Warsaw, Poland, from 25 June to 29 August 2025. The study was non-invasive and relied solely on anonymous self-report questionnaires. It was carried out in accordance with the Declaration of Helsinki. Ethics approval was obtained from the Medical University of Warsaw Bioethics Committee: AKBE/141/2025.

### Participants

Adults with a diagnosis of ALS under neurological outpatient care who could provide informed consent and complete questionnaires independently were eligible. Researcher assistance was allowed in cases of motor limitations. We excluded individuals who did not consent, had conditions that precluded reliable self-report, for example acute decompensation or major communication barriers, or had other clinical contraindications. Recruitment used consecutive sampling during routine visits. Only fully completed questionnaire sets were included in the analyses. During the visit, the researcher explained the purpose, voluntariness, and anonymity of participation and obtained informed consent, then handed out the instrument set. Any assistance was limited to reading questions aloud and marking the responses indicated by the participant, without suggestion.

### Measures

We used three instruments: (1) An author-designed sociodemographic and health questionnaire that captured age, sex, education, employment, marital status, place of residence, living conditions, time from first symptoms to diagnosis, site of onset, feeding and breathing method, and current symptoms that limit functioning; (2) HRQoL was assessed with the Polish version of the WHO Quality of Life-BREF [WHOQOL-BREF; WHO version (15)]. It contains 26 items across four domains: physical, psychological, social relationships, and environment. Responses referred to the last 4 weeks. Domain scores were transformed to a 0–100 scale according to WHO guidance; (3) illness acceptance measured with the AIS, originally developed by Felton, Revenson and Hinrichsen and adapted for Poland by Juczyński (16). The AIS comprises eight statements describing negative consequences of poor health (e.g., limitations, lack of self-sufficiency, dependence on others, reduced self-esteem), rated on a 5-point scale (1 = strongly agree to 5 = strongly disagree). Item scores are summed to yield a total of 8–40, with higher scores indicating greater acceptance (i.e., lower psychological discomfort). AIS total was treated as a continuous variable in correlation and regression analyses. We followed the official Polish scoring rules from the instrument manual.

### Statistical analysis

Continuous variables were summarized as mean ± SD and, where appropriate, as median with interquartile range (IQR). Categorical variables were presented as counts (n) and percentages (%). Distributional normality of continuous outcomes, that is WHOQOL-BREF domain scores and AIS total, was examined with the Shapiro–Wilk test and showed no substantial departures. Given modest subgroup sizes and unequal group counts we preferentially used rank-based tests for between-group comparisons. For comparisons across clinical subgroups, for example feeding method, respiratory status, housing conditions, we used Kruskal–Wallis rank ANOVA (and Mann–Whitney U tests for two-group comparisons) with appropriate *post hoc* pairwise tests. Correlations were assessed with Spearman’s *ρ*. For multivariable analyses, we fitted ordinary least squares (OLS) linear regression models with HC3 heteroskedasticity-consistent robust standard errors. Given sample size constraints, the primary adjusted models included AIS with age and sex as prespecified covariates; we avoided over-parameterization and interpreted all effects as associative rather than causal. For multiple exploratory comparisons, we controlled the false discovery rate (FDR) using the Benjamini–Hochberg procedure. Specifically, for the exploratory between-group comparisons of AIS across sociodemographic and clinical variables (excluding multiple-response items), we applied Benjamini–Hochberg FDR correction across the set of global tests (11 variables) and report both raw *p*-values and FDR-adjusted q-values. Model diagnostics included inspection of residual patterns. Missing data were handled with complete-case analysis (CCA). Statistical analyses were performed in Statistica 13.0 (StatSoft, Tulsa, OK, United States). Two-tailed *p*-values <0.05 were considered statistically significant.

## Results

### Study population characteristics

We analyzed 45 patients with ALS (mean age 52 ± 14 years); women accounted for 58% of the sample. Full sociodemographic and health characteristics are shown in Table 1. In the WHOQOL-BREF, mean scores were: physical domain 46.9 ± 14.1, psychological 51.2 ± 16.9, social relationships 53.0 ± 24.6, and environment 58.4 ± 18.4. The mean AIS score was 20.4 ± 8.1 (Table 2).

**Table 1:** General characteristics of the study population.

Variable	Category	*n*	%
Sex	Female	26	57.8
	Male	19	42.2
Marital status	Single	9	20.0
	Widowed	9	20.0
	Married	27	60.0
Education	Primary	3	6.7
	Higher	17	37.8
	Vocational	12	26.7
	Secondary	13	28.9
Employment	Retirement pension	12	26.7
	Disability pension	16	35.6
	Part-time	8	17.8
	Full-time	9	20.0
Place of residence	Large city	20	44.4
	Small town	21	46.7
	Village	4	8.9
Socioeconomic living conditions	Very good	12	26.7
	Good	20	44.4
	Average	13	28.9
Household composition	Living alone	10	22.2
	With children	6	13.3
	With spouse	16	35.6
	With spouse and children	10	22.2
	With parents	3	6,7
Time from first symptoms to diagnosis	< 1 year	9	20.0
	1–2 years	14	31.1
	2–3 years	10	22.2
	> 3 years	12	26.7
First symptoms (multiple response)	Muscle cramps or twitching	19	42.2
	Weakness of upper and/or lower limbs	31	68.9
	Swallowing problems	12	26.7
	Weight loss	16	35.6
	Speech change and articulation problems	14	31.1
Breathing	Intermittent dyspnea	15	35.7
	Normal	13	31.0
	Respiratory support, NIV	14	33.3
Current symptoms limiting functioning (multiple response)	Difficulties with mobility (for example walking, climbing stairs, changing position in bed)	27	60.0
	Swallowing difficulties	11	24.4
	Excessive salivation	10	22.2
	Sleep problems	15	33.3
	Speech difficulties	21	46.7
	Breathing difficulties	17	37.8
	Manual tasks (for example writing, meal preparation, dressing)	22	48.9
Assistive/rehabilitation equipment available	Any device	40	88.9
	Cervical collar	1	2.2
	Cane(s)	18	40.0
	Orthosis	2	4.4
	Wheelchair	19	42.2

**Table 2:** WHOQOL-BREF and AIS-overall results.

Variable	Mean	SD	95% CI (lower)	95% CI (upper)
WHOQOL-BREF: physical domain (0–100)	46.9	14.1	42.7	51.1
WHOQOL-BREF: psychological domain (0–100)	51.2	16.9	46.1	56.3
WHOQOL-BREF: social relationships domain (0–100)	52.96	24.6	45.6	60.3
WHOQOL-BREF: environment domain (0–100)	58.4	18.4	52.9	63.9
AIS (8–40)	20.4	8.1	17.9	22.8

### Sociodemographic and clinical variables and their relationships with HRQoL and AIS

Among clinical factors, feeding method (self-reported primary/current route: PEG vs. oral feeding) showed the strongest differentiation: individuals with PEG had significantly lower WHOQOL-BREF scores in the physical and environment domains than those with oral feeding (*p* = 0.008 and *p* = 0.012, respectively), with trends toward lower scores in the psychological and social domains (*p* = 0.077 and *p* = 0.100). A similar profile appeared for breathing status. Comparison of three states (normal, intermittent dyspnea, NIV) showed significant differences in the psychological domain (H = 8.643, *p* = 0.013) and the physical domain (H = 6.559, *p* = 0.038), with no significance in the social and environment domains (*p* = 0.141 and *p* = 0.140). Better living conditions were linked to higher quality of life in the psychological and environment domains (H = 11.659, *p* = 0.003 and H = 14.579, *p* < 0.001), with a trend in the physical domain (H = 5.113, *p* = 0.078). Demographic variables showed weaker associations. For example, sex did not differentiate any domain (*p* > 0.05). Time from first symptoms to diagnosis did not correlate with WHOQOL-BREF domains, but showed a positive correlation with AIS (r = 0.37, *p* = 0.014). To further characterize illness acceptance, AIS was examined across sociodemographic and clinical factors (Table 3) with FDR control applied across variables. After FDR correction, only time from first symptoms to diagnosis remained significantly associated with AIS (*p* = 0.001; FDR q = 0.007). AIS was highest in participants reporting >3 years to diagnosis (median 26.5) and lowest in those reporting 2–3 years (median 12.5), suggesting heterogeneity in adaptation trajectories rather than a simple linear more time–more acceptance pattern. Importantly, this association should be interpreted cautiously in a cross-sectional design, as it may reflect confounding by disease trajectory (e.g., slower progression) or unmeasured psychosocial factors rather than any beneficial effect of delayed diagnosis. Other factors including sex, marital status, education, employment, place of residence, household composition, feeding method, breathing status, and assistive equipment availability were not significantly associated with AIS after FDR correction. Socioeconomic living conditions showed a nominal association with AIS (*p* = 0.029), with higher acceptance in the ‘very good’ group; however, this effect did not remain significant after FDR adjustment (q = 0.162) and should be considered exploratory. AIS did not significantly differ across education categories (*p* = 0.434; q = 0.764) or household composition groups (*p* = 0.660; q = 0.803), and the apparent lower AIS among those with higher education should be interpreted cautiously given small cell sizes (notably primary education, *n* = 3).

**Table 3:** Illness acceptance (AIS) across sociodemographic and clinical factors.

Variable	Category	AIS median (IQR)	*p*	FDR q
Sex	Female	20 (13.25–25)	0.730	0.803
	Male	20 (16–26)		
Marital status	Single	17 (15–20)	0.541	0.764
	Widowed	20 (15–25)		
	Married	21 (15.5–26.5)		
Education	Primary	25 (23–26.5)	0.434	0.764
	Vocational	21 (14–26.25)		
	Secondary	20 (17–25)		
	Higher	17 (10–23)		
Employment	Retirement pension	21 (18–26)	0.434	0.764
	Disability pension	19.5 (10.8–24.8)		
	Part-time	17 (14.5–19.25)		
	Full-time	21 (15–28)		
Place of residence	Large city	21.5 (17–28)	0.264	0.764
	Small town	18 (14–24)		
	Village	16 (11–21.5)		
Socioeconomic living conditions	Very good	26.5 (20–30)	0.029	0.162
	Good	19.5 (17–23.25)		
	Average	14 (11–21)		
Household composition	Living alone	18.5 (13.5–24.8)	0.660	0.803
	With children	16.5 (15–19.5)		
	With spouse	24 (16.3–28.3)		
	With spouse and children	19.5 (14.75–21)		
	With parents	21 (19–24.5)		
Time from first symptoms to diagnosis	< 1 year	19 (15–21)	<0.001	0.007
	1–2 years	18 (13.5–20)		
	2–3 years	12.5 (9.3–19.3)		
	> 3 years	26.5 (23.8–30.8)		
Feeding method	Oral feeding	20 (15–26)	0.345	0.764
	PEG	17.5 (11.8–23.3)		
Breathing	Normal	20 (17–28)	0.555	0.764
	Intermittent dyspnea	20 (15–24.5)		
	NIV	17.5 (10.3–26.8)		
Assistive/rehabilitation equipment	Yes	20 (14.8–26)	0.957	0.957
	No	20 (19–21)		

### Association of AIS with HRQoL

Illness acceptance correlated positively with each WHOQOL-BREF domain. Spearman’s r ranged from 0.40 to 0.52, all *p* ≤ 0.006 (Table 4). In linear regression models, AIS remained an independent correlate of domain scores: physical *β* = 0.96, *p* = 0.003; psychological β = 0.94, *p* = 0.013; social relationships β = 1.47, *p* = 0.003; environment β = 1.10, *p* = 0.025 (Table 5). Practically, a 10-point increase in AIS was associated with an improvement of about 9 to 15 points on the 0–100 scale, depending on the domain.

**Table 4:** Association of AIS with WHOQOL-BREF domains.

Domain	R	*P*
Physical	0.518	<0.001
Psychological	0.464	0.001
Social relationships	0.402	0.006
Environment	0.503	<0.001

**Table 5:** Acceptance of illness and quality of life in ALS: age and sex adjusted models.

Domain (0–100)	Covariate	β	SE (HC3)	95% CI (β)	*p*
Physical	Intercept	40.39	9.39	21.99 to 58.80	<0.001
	AIS (per 1 point)	0.96	0.32	0.33 to 1.59	0.003
	Age (per 1 year)	−0.24	0.14	−0.51 to 0.04	0.093
	Sex: male vs. female	3.49	3.99	−4.33 to 11.31	0.382
Psychological	Intercept	46.05	13.58	19.43 to 72.67	<0.001
	AIS (per 1 point)	0.94	0.38	0.20 to 1.68	0.013
	Age (per 1 year)	−0.25	0.20	−0.64 to 0.14	0.206
	Sex: male vs. female	4.53	5.21	−5.69 to 14.75	0.385
Social	Intercept	36.21	22.23	−7.36 to 79.78	0.103
	AIS (per 1 point)	1.47	0.49	0.52 to 2.43	0.003
	Age (per 1 year)	−0.30	0.32	−0.94 to 0.33	0.349
	Sex: male vs. female	12.55	8.39	−3.89 to 28.98	0.135
Environmental	Intercept	29.36	12.08	5.68 to 53.04	0.015
	AIS (per 1 point)	1.10	0.49	0.14 to 2.06	0.025
	Age (per 1 year)	0.16	0.18	−0.20 to 0.52	0.382
	Sex: male vs. female	4.96	5.18	−5.20 to 15.12	0.339

## Discussion

### HRQoL profile in the study sample

The HRQoL pattern observed in our cohort, as assessed with WHOQOL-BREF (Table 2), showed the greatest impairment in the physical domain relative to the other domains, which is consistent with ALS literature indicating that limitations in physical functioning and activity are typically most compromised (17). To provide external context using the same instrument, a large UK benchmark dataset of WHOQOL-BREF administered across diverse diseases and conditions reported that physical-domain scores in the mid-40s are observed in high-burden musculoskeletal conditions such as arthritis (mean 46.45), whereas endocrine/metabolic conditions such as diabetes tend to report substantially higher physical-domain scores (mean 67.84). In the same dataset, healthy samples generally scored in the high-60s to mid-70s across domains (e.g., “Well” group: Physical 76.49; Environment 68.20), but the authors emphasize that these benchmarks are pragmatic rather than population norms because the sample was not randomized nor fully representative (18). Against this comparative frame, the pattern in our ALS cohort is compatible with a disease profile in which progressive functional limitations are likely to disproportionately constrain the physical domain, while other QoL domains may be partially preserved. Importantly, ALS disease severity/stage is expected to modify WHOQOL-BREF profiles: studies in ALS indicate that WHOQOL-BREF domains- particularly the physical domain- show a strong gradient across ALSFRS-R levels. In support of this, a systematic review of qualitative/functional disability and QoL measures in ALS reports that WHOQOL-BREF domain scores show significant differences and a strong gradient across most ALSFRS-R disability levels, with the physical domain demonstrating particularly marked stage separation (19). Therefore, stage should be treated as a major source of between-patient variability when interpreting domain profiles and when comparing cohorts.

### Illness acceptance and the context of HRQoL measurement

AIS correlated positively with each WHOQOL-BREF domain. For external context using the same AIS + WHOQOL-BREF pairing, in multiple sclerosis Rosińczuk et al. (20) reported strong positive correlations between AIS and WHOQOL-BREF domains (Physical r = 0.75; Psychological r = 0.67; Social r = 0.66; Environment r = 0.66), with mean AIS 24.83 (SD 9.41). Similarly, in ankylosing spondylitis Wysocki et al. (21) found positive associations between AIS and WHOQOL-BREF domains, with the strongest association in the physical domain (rho = 0.71) and the weakest in the social domain (rho = 0.329), illustrating domain-specific attenuation that is particularly relevant when interpreting social-relationships scores. Importantly, from a severity/stage perspective, acceptance was lower among participants with a certificate of disability (AIS 23.68 ± 7.89 vs. 29.43 ± 8.50; *p* < 0.001), suggesting a disability/severity signal for AIS in at least some long-term conditions, although this proxy is not equivalent to ALS clinical staging and should be interpreted cautiously (20, 21). Against this external context, our ALS findings indicate small-to-moderate positive associations (Table 4), suggesting that acceptance captures a meaningful but not exclusive component of HRQoL variability in ALS. Cross-study comparisons of correlation magnitudes should be interpreted cautiously, because they are sensitive to range restriction, disease-severity mix, administration mode, and unmeasured psychological comorbidity. In ALS specifically, AIS–stage evidence remains limited and, in our dataset, the absence of ALSFRS-R or formal staging precludes direct testing of whether AIS varies systematically across disability strata; therefore, any stage-related interpretation for AIS should be framed as a hypothesis rather than a conclusion. Moreover, AIS items partly reflect dependence and reduced self-sufficiency, which may overlap conceptually with physical functioning and could influence domain-specific associations (21). Accordingly, it is plausible that a generic acceptance measure captures only part of ALS-relevant appraisal and meaning-making, but this remains a hypothesis that requires longitudinal and ALS-specific psychometric work. In OLS models adjusted for age and sex, AIS remained an independent correlate of domain scores, which aligns with evidence that psychological components meaningfully differentiate HRQoL (22). Our findings highlight a clinically meaningful dissociation between symptom-related impairment and psychological adaptation. On the one hand, clinical status variables (feeding method, respiratory status) and living conditions differentiated WHOQOL-BREF domains, consistent with the direct functional and environmental constraints imposed by ALS. On the other hand, AIS displayed limited between-group differences across most sociodemographic and clinical strata after FDR correction, despite remaining a consistent independent correlate of all WHOQOL-BREF domains in adjusted models. This apparent divergence supports the interpretation that illness acceptance captures an internal coping and meaning-making dimension that may not track linearly with objective disease milestones at a single time point. The robust association between AIS and time from first symptoms to diagnosis should be interpreted cautiously: it may reflect heterogeneity in disease trajectories, survivorship and selection effects, or unmeasured psychosocial resources rather than any beneficial impact of diagnostic delay. Longitudinal studies integrating progression rate and targeted psychological measures are needed to clarify whether acceptance evolves as a function of time, symptom change, or supportive interventions. Our findings are consistent with research on cognitive adaptation to disease. Kruitwagen-van Reenen et al. (9) analyzed three dimensions of the Illness Cognitions Questionnaire (ICQ) and their links to HRQoL measured with the ALS Assessment Questionnaire-40 (ALSAQ-40). Higher helplessness was associated with poorer HRQoL. Helplessness increased as function declined on the ALS Functional Rating Scale—Revised (ALSFRS-R). The authors argued for early identification of patients with high helplessness and low acceptance and for monitoring these dimensions over time, especially before PEG and NIV decisions. This pattern is consistent with a conceptual model in which acceptance may contribute to the association between disease-related impairment and HRQoL; however, mediation cannot be inferred in a cross-sectional design. Longitudinal testing with explicit severity/stage measures (e.g., ALSFRS-R or clinical staging) is required to evaluate these pathways (19). Similarly, Gentili et al. (23) found that HRQoL in ALS was driven most by psychological and social factors rather than by the physical component alone.

Measurement validity findings support this interpretive framework by demonstrating convergent validity between WHOQOL-BREF and ALS-specific HRQoL instruments, and by confirming that WHOQOL-BREF domains discriminate disability severity in ALS. The ALS-Specific Quality of Life–Short Form (ALSSQOL-SF) showed good reliability and acceptable fit of a six-dimensional model in confirmatory factor analysis. Its correlation with WHOQOL-BREF was high at r = 0.745, indicating strong convergence between a disease-specific and a generic measure and supporting our choice of WHOQOL-BREF (24). In practice, the brief WHOQOL-BREF can be combined with the disease-specific ALSSQOL-SF when greater sensitivity to ALS-specific aspects, such as bulbar functions or intimacy, is needed.

Validation of WHOQOL-BREF in ALS was confirmed in the Trajectories of Outcomes in Neurological Conditions (TONiC) study with 636 patients. Rasch model fit was shown for the physical, psychological, and environment domains, with no differential item functioning by age, sex, or site of onset. The social domain had weaker properties as a stand-alone scale but supports the overall score. WHOQOL-BREF domains differentiated disability levels per ALSFRS-R, and associations with the WHO Disability Assessment Schedule 2.0 (WHODAS 2.0), modified Hospital Anxiety and Depression Scale, and EQ-5D-5L were moderate to strong (17). In a second TONiC analysis, the Wilson and Cleary model was reconstructed using Rasch-transformed scores. Activity limitations measured by WHODAS had the strongest effect on HRQoL, followed by anxiety. The analysis suggests that fatigue and dyspnea lower HRQoL mainly indirectly through worse functioning in daily activities. Notably, the dependency structure was stable by sex and site of onset, which implies similar mechanisms across these subgroups. The main differences in WHOQOL-BREF between onset types involved the physical and environment domains. This matches our lowest score in the physical domain and the important role of respiratory symptoms. Together with the AIS signal, it suggests that illness acceptance may modify the pathway from symptoms and activity limitations to HRQoL (8).

In ALS, functional decline is not uniformly mirrored by proportional (linear) declines in self-reported well-being; the association depends on the specific construct assessed (e.g., global QoL vs. depressiveness) and may be non-linear in some patient subgroups (25, 26). Longitudinal data indicate that progression rate and shorter time since symptom onset (both implying limited time to adapt) are associated with lower wellbeing, whereas some QoL indices can remain stable despite measurable ALSFRS-R decline- consistent with response-shift mechanisms. However, this should not be misread as stage/severity being irrelevant: disability level shows a clear gradient in WHOQOL-BREF domains- particularly physical (and also environment), while psychosocial adaptation and response shift may help preserve global/subjective QoL even in advanced disease (19). The so-called well-being paradox reflects psychosocial adaptation and a shifting HRQoL reference point. Interprofessional team support can strengthen this process. A practical frame is the four principles of healthcare ethics. Beneficence calls for early implementation of interventions that improve quality of life, such as NIV, adequate nutritional support, and symptom control, while reinforcing family resources and psychological resilience. Non-maleficence requires careful disclosure of diagnosis, advance care planning, and tailoring information to patient needs to reduce emotional burden. Autonomy highlights systematic communication support, from letter boards and eye tracking to brain-computer interfaces, so patients can express preferences even in advanced stages. Justice underscores equal access to palliative care, assistive technologies, and services regardless of residence or socioeconomic status. This ethical framing provides an interpretive lens for the observed association between higher illness acceptance and better HRQoL in our cohort, rather than evidence of a causal mechanism. AIS operationalizes self-reported acceptance of illness and may serve as a proxy for selected facets of psychological adaptation; however, whether acceptance buffers the impact of disability on HRQoL, or whether care aligned with beneficence, non-maleficence, autonomy, and justice improves acceptance and downstream HRQoL, cannot be inferred from our cross-sectional design and should be tested in longitudinal and interventional studies (27).

### Determinants and clinical–environmental implications

In our sample, PEG feeding was associated with lower HRQoL in the physical and environment domains compared with oral feeding. Respiratory status differentiated HRQoL in the physical and psychological domains. Better living conditions were linked to higher scores in the psychological and environment domains. This profile agrees with reports that, alongside psychological components, functional limitations and environmental factors shape HRQoL. In the area of communication, we observed a trend toward poorer social domain scores in those with speech difficulties. Evidence shows that bulbar phenotype lowers EQ VAS and ALSAQ-5 and worsens social functioning (1). Findings on theory-of-mind deficits and their link with mental well-being justify early speech–language therapy and augmentative and alternative communication support (22, 28). Data from the COVID-19 period indicate that pressure on care systems and social isolation reduced QoL components and perceived adequacy of care, underscoring the sensitivity of outcomes to environmental factors (23). The literature also shows that higher caregiver burden can inversely relate to patient HRQoL, and caregiver-focused interventions improve well-being for both parties (17). In practice, somatic interventions should be paired with acceptance-promoting modules and strengthening of environmental resources. This approach aligns with NICE and EAN frameworks and with COSMIN recommendations for selecting and interpreting PROMs (4). Overall, HRQoL in ALS remains moderately reduced, with the greatest burden in the physical domain. Illness acceptance is an independent correlate across all WHOQOL-BREF domains. Outpatient care should combine reduction of somatic burden with psychological interventions and environmental support.

This direction is consistent with comparative WHOQOL-BREF profiles across multiple long-term conditions, which suggest that substantial physical burden can co-occur with partially preserved non-physical domains, rather than demonstrating specific underlying resources as a mechanism (18). However, convergent evidence for an acceptance-related explanation is available from studies co-administering AIS and WHOQOL-BREF in other long-term conditions (e.g., multiple sclerosis and ankylosing spondylitis), where higher acceptance is strongly associated with better WHOQOL-BREF domain scores. Beyond observational associations, acceptance-based psychological intervention has trial-level evidence in motor neuron disease: in the COMMEND multicentre randomised controlled trial, Acceptance and Commitment Therapy plus usual care improved quality of life measured with the MQOL-R at 6 and 9 months compared with usual care alone (29). Accordingly, acceptance- and coping-oriented support may be considered as an adjunct to symptom management and environmental/assistive interventions, tailored to ALS/MND-related disability and communication needs; however, effects on WHOQOL-BREF domains and cost-effectiveness require further targeted evaluation (30).

### Limitations

This was a cross-sectional study based solely on self-report, which limits causal inference and raises the risk of common-source variance. It was single-center, with a short recruitment window and moderate sample size. We did not include objective indicators of disease severity, such as ALSFRS-R, or standardized measures of comorbid burdens, for example depression, anxiety, and pain. Because we did not capture ALSFRS-R or a formal clinical staging system, we could not quantify stage-specific gradients in WHOQOL-BREF or test whether AIS varies around key disease milestones; therefore, disease stage may confound or modify acceptance–HRQoL associations.

The lack of longitudinal observation precludes assessment of the stability of the observed relationships and their sensitivity to change. Because AIS has not been comprehensively validated in ALS according to COSMIN standards, our findings should be interpreted as associations based on a generic acceptance measure rather than definitive evidence about ALS-specific acceptance measurement performance. Future work should evaluate AIS in ALS using COSMIN-guided psychometric analyses (e.g., structural validity, reliability, measurement invariance, and responsiveness).

## Conclusion

The level of illness acceptance is a consistent and independent correlate of all WHOQOL-BREF domains. Strengthening psychological adaptation should be an equal goal of care alongside symptom management. While education level and household composition were not statistically associated with AIS in this cohort, these contextual factors remain clinically relevant for tailoring communication, caregiver engagement, and access to resources; future studies should test whether they moderate the AIS–HRQoL relationship.

Dysphagia and respiratory problems have the strongest negative impact on HRQoL. Decisions on PEG and initiation of NIV should be routinely paired with speech–language therapy, dietetic care, and psychological support. Environmental factors matter as much as clinical ones. Standards of care should therefore include coordination of social services, home adaptation, and access to assistive communication technologies.

From an organizational standpoint, an integrated package is justified. It should combine pro-adaptive modules that raise illness acceptance with intensified functional rehabilitation. In outpatient practice, the AIS and WHOQOL-BREF domain scores can serve as quality indicators and decision points.

Future studies should test AIS as a mediator of the effects of somatic and environmental interventions on HRQoL. Longitudinal observation is needed around critical milestones, for example PEG placement and NIV initiation. In parallel, the psychometric properties of the instruments used should be verified in the ALS population. A strategy with three pillars, that is reinforcement of psychological adaptation, intensive management of high-impact symptoms, and systematic environmental support, has the greatest potential to improve HRQoL.

## Data Availability

The raw data supporting the conclusions of this article will be made available by the authors, without undue reservation.
